# Clinical performance of Anyplex II HPV28 by human papillomavirus type and viral load in a referral population

**DOI:** 10.1371/journal.pone.0210997

**Published:** 2019-01-23

**Authors:** Ingrid Baasland, Pål R. Romundstad, Maj Liv Eide, Christine M. Jonassen

**Affiliations:** 1 Department of Public Health and Nursing, Norwegian University of Science and Technology, Trondheim, Norway; 2 Department of Pathology, St. Olav’s hospital, Trondheim University Hospital, Trondheim, Norway; 3 Faculty of Chemistry, Biotechnology and Food Science, Norwegian University of Life Sciences, Ås, Norway; 4 Center for Laboratory medicine, Østfold Hospital Trust, Grålum, Norway; Hôpital Bichat-Claude Bernard, FRANCE

## Abstract

Anyplex II HPV28 (`Anyplex`) is a semi-quantitative DNA PCR assay divided into set A, comprising 14 high risk (hr)HPV types; and set B, comprising 5 possibly hrHPV types and 9 low risk (lr)HPV types. We compared the ability of Anyplex to that of Hybrid Capture 2 (HC2) and PreTect HPV-Proofer (`Proofer`) to detect cervical intraepithelial neoplasia grade two or worse (CIN2+) by HPV types and viral load. This cross-sectional study included 296 women referred to colposcopy with abnormal cervical cytology and/or persistent HPV infection. CIN2+ was identified in 175/296 women. Liquid based cytology samples were used to perform HPV testing. The sensitivity of Anyplex to detect CIN2+ was 98.9% (95% CI 95.9–99.9) and specificity 43.0% (95% CI 34.0–52.3). Restricting to medium and high viral loads in Anyplex set A, sensitivity and specificity were 97.1% (95% CI 93.5–99.1) and 59.5% (95% CI 50.2–68.3) with positive (PPV) and negative predictive value (NPV) 77.6% and 93.5%, respectively, comparable to HC2. Restricting Anyplex to the hrHPV types in Proofer, HPV16, 18, 31, 33 and 45, sensitivity and specificity for CIN2+ were 85.1% (95% CI 79.0–90.1) and 71.1% (95% CI 62.1–79.0), comparable to Proofer`s. When adding HPV52 and 58, the sensitivity for CIN2+ was 92.6% (95% CI 87.6–96.0) and CIN3+ 96.5% (95% CI 92.0–98.8). No value of Anyplex set B was found in detecting CIN2+. In conclusion, the clinical performance of medium and high viral loads in Anyplex set A was comparable to HC2. Restricting the test to the 7 hrHPV types included in the 9-valent HPV-vaccine, HPV16, 18, 31, 33, 45, 52 and 58, satisfies the international criteria for cervical cancer screening with relative sensitivity compared to HC2 for CIN2+ and CIN3+ of 0.98 and 1.01, respectively. Detecting all 28 Anyplex HPV types adds no benefit in a referral population.

## Introduction

Human papillomavirus (HPV) testing is frequently used in triage, follow-up and increasingly used in primary cervical cancer screening. HPV genotyping has focused on HPV 16 and 18, based on their contribution to cervical cancer. But in a screening perspective, the aim is to prevent cancers by detecting precursor lesions, where other types are highly important. Most commercial HPV tests targeting high risk (hr) HPV types are detecting the 12 carcinogen HPV types 16, 18, 31, 33, 35, 39, 45, 51, 52, 56, 58 and 59), in addition to HPV 68, classified as probably carcinogenic, and HPV 66, a possibly carcinogenic type [1]. Risk stratification for cervical intraepithelial neoplasia grade 3 or worse (CIN3+) by extended HPV genotyping may be useful in clinical management and in a screening setting [2, 3]. An international expert panel suggested in 2009 guidelines for evaluating hrHPV DNA tests for primary screening based on their sensitivity and specificity according to the clinically validated reference Hybrid Capture 2 (HC2) for the detection of CIN2+[4, 5], one criterion being a relative sensitivity for CIN2+ compared with HC2 >90%. Most HPV genotyping assays are time-consuming and require some degree of expertise, making them less useful in clinical practice. Recently, a semi-quantitative, real-time multiplex PCR assay called Anyplex II HPV28 (hereafter referred to as Anyplex; Seegene, Seoul, South Korea) became available. The test is divided into two sets: set A comprises 14 hrHPV types; and set B comprises 5 possibly carcinogenic/high risk (phr)HPV types and 9 low risk (lr)HPV types. Studies have concluded that Anyplex requires less time [6] and less expertise than most other HPV genotyping assays [7], while still being very sensitive and specific [7–10].

The need for better risk stratification of HPV DNA-positive women increases as the positive predictive value (PPV) of any HPV test, both in terms of HPV positivity and identifying women with CIN2+, will be affected by the decreasing prevalence in women who undergo repeated HPV screening. In addition, genotyping tests might prove useful in the screening of vaccinated women.

The full genotype information provided by Anyplex allows for immediate risk stratification based on genotype and viral loads, and might prove highly useful in screening, follow-up and in a post-treatment setting. In this study, we compared the ability of Anyplex to that of the DNA test HC2 (Digene, formerly Qiagen Corporation, Gainthersburg, MD, USA) and the highly clinically specific mRNA test PreTect HPV-Proofer (hereafter referred to as Proofer; Norchip, Klokkarstua, Norway) to detect CIN2+ by HPV type and viral loads in a population with high HPV prevalence population. In particular, we looked at the following five specific groups of HPV types in Anyplex: 1) HPV16 and 18, 2) the five hrHPV types in Proofer, 3) the seven hrHPV types in the 9-valent HPV vaccine Gardasil9, 4) the HPV types in Anyplex set A, and 5) the HPV types in set A and B.

## Materials and methods

### Study sample

The study population and data collection has been described previously [11]. Briefly, the study population consisted of 305 consecutive patients with abnormal cervical cytology results and/or persistent HPV infection [12] referred to colposcopy and biopsy between November 2010 and June 2012 at the Department of Obstetrics and Gynecology, St. Olav’s University Hospital. The guidelines for referral to colposcopy included either high-grade cytological results, or abnormal cytology and/ or HPV positivity at follow-up 6–12 months after primary low-grade cytological results. At follow-up, liquid-based cytology (LBC) samples were taken with plastic spatula-cytobrush, placed in PreservCyt solution (Hologic, Bedford, MA, USA) according to the manufacturer’s instructions, and used for Anyplex, HC2, and Proofer. Each woman also had colposcopy-directed biopsies and endocervical curettage taken; some had cone specimens taken. These samples were fixed in formalin and embedded in paraffin blocks.

**Fig 1:**
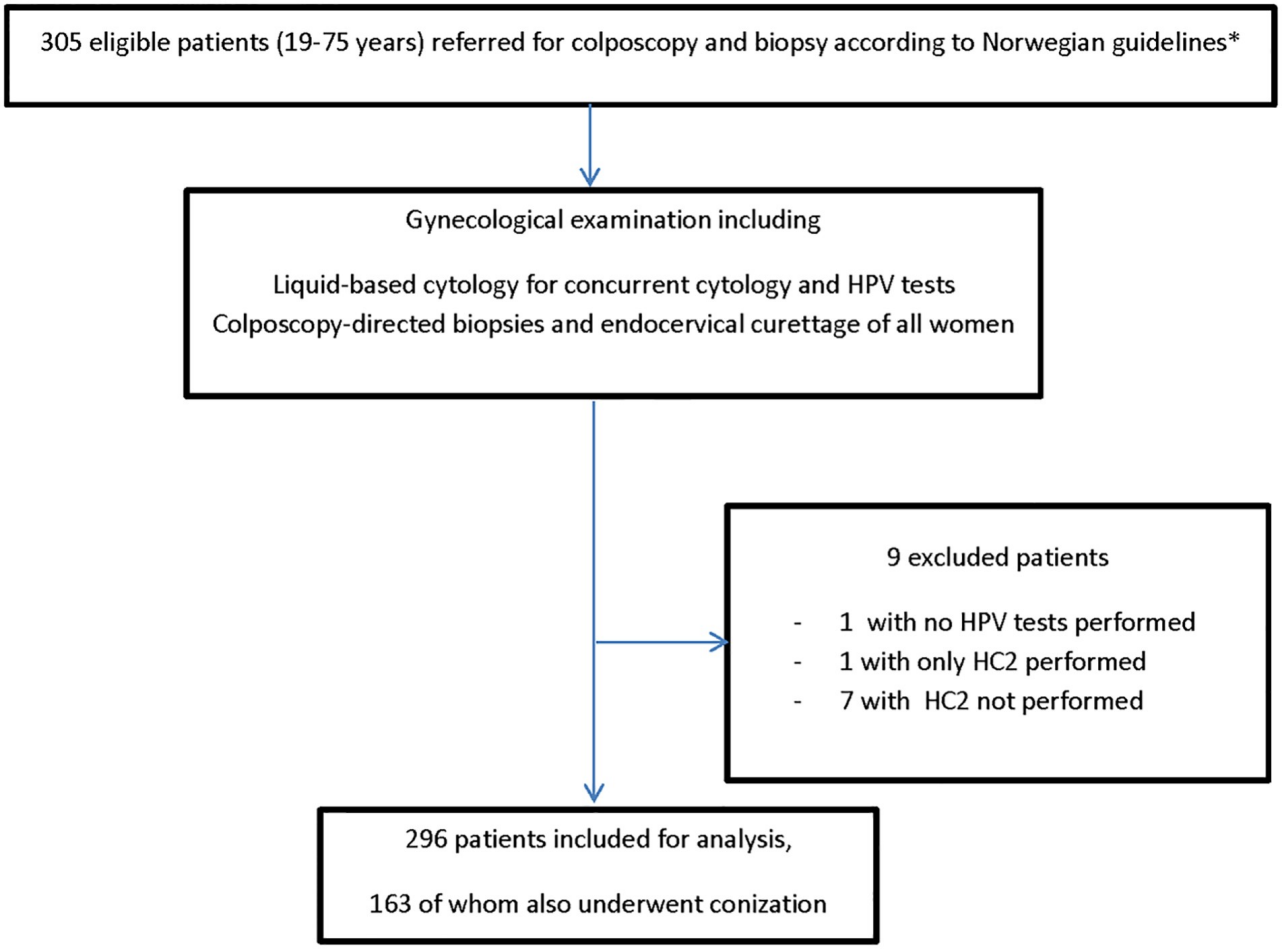
Flow chart of the study. *Norwegian guidelines for histological examination were persistent low-grade squamous intraepithelial lesion or atypical squamous cells of undetermined significance+high-risk HPV, persistent high-risk HPV-positive cervical smears, atypical squamous cells, cannot rule out a high-grade lesion, high-grade squamous intraepithelial lesion and adenocarcinoma in situ. Abbreviations: HPV: human papillomavirus, HC2: hybrid capture 2.

Following HPV testing, we excluded nine patients who did not have results for all three HPV tests, leaving 296 patients for the current analysis (Fig 1). Written patient information was given, and written informed consent was obtained from all study participants. We followed the STARD reporting guidelines, and the study was approved by the Regional Committee for Ethics in Medical Research, West Region, Norway (2010/420).

### HPV testing

All HPV tests were performed within the framework of a collaboration between the laboratory of St. Olav’s University Hospital and Norwegian University of Science and Technology.

HC2 was performed on cell sample aliquots according to the manufacturer’s instructions. HC2 detects 13 hrHPV types (HPV16, 18, 31, 33, 35, 39, 45, 51, 52, 56, 58, 59, and 68). The test does not include genotyping, thus a positive test refers to detection of one or more of the included HPV types. The test has no internal sample control. The residual cell suspensions in LBC samples were stored at 4–8°C for up to 12 weeks before further processing.

Nucleic acid extraction for Proofer and Anyplex testing was performed using the semiautomatic NucliSens miniMAG (BioMerieux SA, Marcy I’Etoile, France) protocol, which has been validated for both DNA and mRNA tests [13]. A cell aliquot was pelleted from the PreservCyt medium by centrifugation and suspended in lysis solution. Extraction was performed from the whole lysate, with a final elution volume of 50 μl. Isolated nucleic acid was used for Proofer testing according to the manufacturer’s instructions. This type-specific mRNA test detects E6/E7 full-length mRNA transcripts of the five hrHPV types most commonly associated with cervical cancer worldwide: HPV16, 18, 31, 33, and 45 [14]. Proofer has previously been shown to have lower sensitivity, but higher specificity than HC2 to detect CIN2+ [13, 15, 16]. The test does have an internal mRNA control.

The rest of the isolated nucleic acid was stored at -80°C until Anyplex testing was performed retrospectively in 2013, once all women had been recruited, according to the manufacturer’s instructions. Anyplex is a HPV DNA genotyping test that can simultaneously detect, differentiate, and semi-quantify 28 HPV genotypes (19 hrHPV types and 9 lrHPV types) in only two PCR wells per sample. The test is divided into two sets: set A comprises 14 hrHPV types (HPV16, 18, 31, 33, 35, 39, 45, 51, 52, 56, 58, 59, 66, and 68), and set B comprises 5 phrHPV types (HPV26, 53, 69, 73, and 82) and 9 lrHPV types (HPV 6, 11, 40, 42, 43, 44, 54, 61, and 70). HPV 70 is categorized as a lrHPV type in Anyplex, although it has been classified as a class 2b carcinogen [17]. The test can be used with different extraction methods, but it has a fully-automated DNA extraction and PCR setup option, which simplifies the genotyping of the 28 included HPV types. The test is a multiplex real-time PCR assay utilizing dual priming oligonucleotide and tagging oligonucleotide cleavage and extension technology. Viral load is semi-quantified in Anyplex as high (+++; positive signal before 31 PCR cycles), medium (++; positive signal between 31 and 39 PCR cycles), or low (+; positive signal after 40 PCR cycles), and has an endogen internal control.

Both for Anyplex and Proofer, samples with negative internal control and a negative HPV result, were re-analyzed by the same test. If the internal control was negative, but the test result was positive, the test result was considered valid. Negative tests with a positive internal control were not re-analyzed, neither were samples with negative HC2 results, since this test has no internal control.

### Histology

All endocervical curettage samples and biopsies were reviewed by two pathologists who were not blinded to referral cytology. Histology was classified according to the WHO classification of 2003 [18], with CIN grades 1–3 in the squamous epithelium and adenocarcinoma in situ (ACIS) in the endocervical epithelium. The most severe diagnosis from endocervical curettage, biopsy, or cone specimen, was used in the comparison of the three HPV tests, with CIN2+ and CIN3+ as the endpoints.

### Statistical analysis

Performance of Anyplex, HC2, and Proofer were evaluated according to histology using sensitivity, specificity, PPV, negative predictive value (NPV), and their respective 95% confidence intervals (CIs). Specificity and NPV were reported for <CIN2, since detection of CIN2 was not considered a false-positive result. Agreement between the different HPV assays was calculated by using Cohen’s ĸ as an indicator of concordance; ĸ-values <0.2 indicated slight agreement, 0.21–0.40 weak agreement, 0.41–060 moderate agreement, 0.61–0.80 substantial agreement, and >0.81 almost perfect agreement [19]. Statistical analyses were performed using STATA 14.0 (Stata Corp, College Station, Texas, USA).

## Results

Mean age among the 296 women in the study sample was 35 years (range 19–75). Mean time from referral to follow-up was 98 days (range 13–485; median 91 days). Referral LBC indicated high-grade disease in 77.7% (n = 230) of the study sample, and histology revealed CIN2+ in 59.1% (n = 175), including five patients with ACIS, four of whom were concurrently diagnosed with CIN3, and three patients with microinvasive squamous carcinoma in cone specimens. All three cancers were diagnosed in participants aged 34–49 years and showed CIN3 in punch biopsies.

A total of 245 samples (82.7%) were positive for HPV with at least one HPV test used in the study. Although Anyplex includes a total of 28 HPV types and HC2 includes 13 types, the overall agreement between these assays was substantial (87.8%), with a kappa value of 0.66 (95% CI 0.53–0.75), while the agreement between Anyplex and Proofer, which includes five HPV types, was moderate (73.0%), with a kappa of 0.42 (95% CI 0.32–0.52) (Table 1).

**Table 1:** Concordance among human papillomavirus (HPV) test results in a referral population of 296 Norwegian women.

		Anyplex II HPV28			Agreement	Kappa (95% CI)
		Positive	Negative	Total		
HC2	Positive	209	3^1^	212	87.8%	0.66 (0.53–0.75)
	Negative	33^2^	51	84		
Proofer	Positive	163	1^3^	164	73.0%	0.42 (0.32–0.52)
	Negative	79^4^	53	132		
Total		242	54	296		

^1^Anyplex negative and HC2 positive, n = 3; One false negative Anyplex28 HPV 45, detected in a concordant positive Proofer test. The other two discordant tests had negative Proofer and negative histology

^2^Anyplex positive and HC2 negative, n = 33; including 18 samples with high-risk HPV types, 6 samples with possible high-risk HPV types not included in HC2 and 9 samples with low-risk HPV types. Distribution of the high-risk HPV types: HPV 16 (n = 2), HPV 31 (n = 5), HPV 35 (n = 1), HPV 45 (n = 4), HPV 39 (n = 1), HPV 51 (n = 1), HPV 52 (n = 2), HPV 58 (n = 1), HPV 68 (n = 1) and 6 samples with HPV:

^3^Anyplex negative and Proofer positive, n = 1; one false negative Anyplex where Proofer detected HPV45.

^4^Anyplex positive and Proofer negative, n = 79; including 70 samples with high risk and/or possible high-risk HPV types and 9 samples with low risk HPV types. Distribution of the high-risk HPV types: HPV 16 (n = 3), HPV 18 (n = 5). HPV 31 (n = 10), HPV 33 (n = 3). HPV types not included Proofer (n = 49)

Abbreviations: CI: confidence interval, HC2: hybrid capture 2, Proofer: PreTect HPV-Proofer.

**Fig 2:**
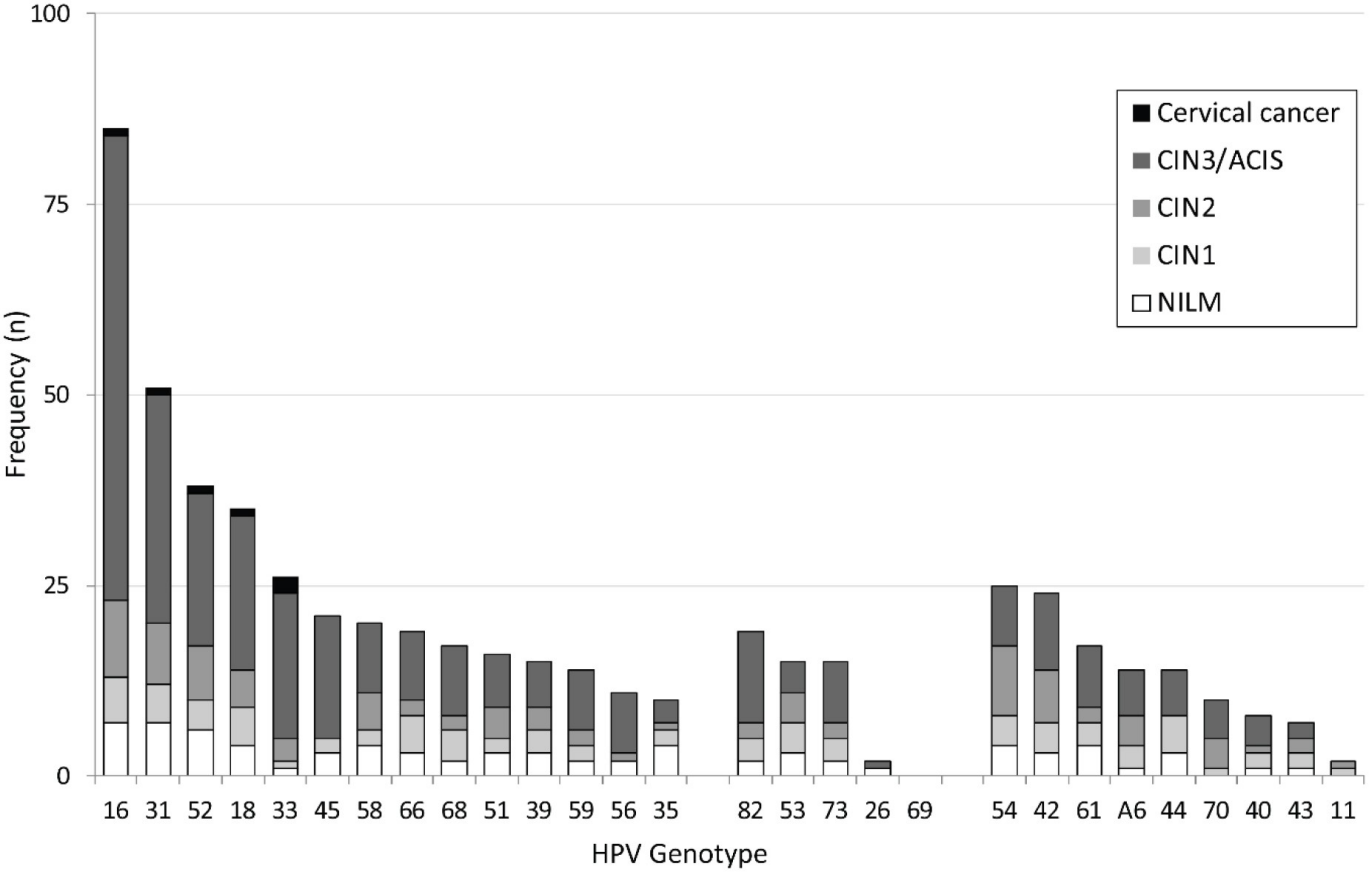
HPV distribution according to histology. Abbreviations: HPV: human papillomavirus, NILM: negative for intraepithelial lesion and malignancy, CIN1: cervical intraepithelial neoplasia grade 1, CIN2: cervical intraepithelial neoplasia grade 2, CIN3: cervical intraepithelial neoplasia grade 3, ACIS: adenocarcinoma in situ.

Based on Anyplex results, HPV16 was the most common type, being present in -28.7% of women, followed by HPV31, 52, 18, 33, and 45 in 17.2%, 12.8%, 11.8%, 8.8%, and 7.1%, of the study sample, respectively. HPV type distribution among women with CIN2+ reflected that of the entire study sample, but with higher positivity rates: 41.1%, 22.3%, 16.0%, 14.9%, 13.7%, and 9.1%, respectively (Fig 2). The incidence of multiple-type HPV infections detected by Anyplex did not differ substantially between women with normal histology, CIN1, and CIN2+; i.e., 40.0% (95% CI 25.7–55.7), 70.8% (95% CI 48.9–87.4), and 56.6% (95% CI 48.9–64.1), respectively. Multiple-type HPV infections were substantially more common among women under 34 years of age (61.7%, 95% CI 53.5–69.4) than in older women (26.7%, 95% CI 19.7–34.8).

Among the 242 women with positive Anyplex results, 95.9% had medium or high viral loads. When looking at only the 175 women with CIN2+, 173 had positive Anyplex, of whom 99.4% had a medium or high viral load (98.2% of all CIN2+ cases). However, when we restricted analyses to cases with high viral load only, the sensitivity of Anyplex to detect the 175 CIN2+ cases decreased from 98.2% to 61.3% (Table 2), reflecting the difference in the diagnostic value of Anyplex to detect CIN2+ by viral load.

**Table 2:** Diagnostic value of viral loads in Anyplex II HPV28 to detect high grade dysplasia.

	CIN2+			
	Medium/high viral load		High viral load	
	n	%	n	%
HPV types included in:				
Anyplex Set A^1^	170	97.1	102	58.3
Set A^1^ + phr HPV types in set B^2^	171	97.7	104	59.4
Set A^1^ + set B^3^	172	98.2	106	61.3

^1^ Including HPV16,18,31,33,35,39,45,51, 52,56,58,59,66,68

^2^ Including HPV26,53,69,73,82

^3^ Including HPV26,53,69,73,82,6,11,40,42,43,44,54,61,70

Abbreviations: CIN2+: cervical intraepithelial neoplasia grade 2 or worse, phr: possible high-risk

Footnote: One CIN2+ had low viral load (HPV42) and two CIN2+ had negative Anyplex

We observed very few false-negatives for CIN2+ by Anyplex and by HC2: 2 (1.1%, 95% CI 0.1–4.1%) and 10 (5.7%, 95% CI 2.7–10.3%), respectively. Among the 242 women with a positive Anyplex result, 69 had CIN1 or normal histology (Table 3), leaving a PPV of 71.5% (95% CI 65.4–77.1) and a NPV of 96.3% (95% CI 87.3–99.5) for high-grade dysplasia. The predictive values of HC2 were comparable, with a slightly higher PPV (77.8%, 95% CI 71.6–83.2), but lower NPV (88.1%, 95% CI 79.2–94.1). In contrast, substantially different predictive values were seen for Proofer, with a higher PPV (84.8%, 95% CI 78.3–89.9) and lower NPV (72.7%, 95% CI 64.3–80.1).

**Table 3:** Histology related to human papillomavirus (HPV) detection in a referral population of 296 Norwegian women.

	Positivity rate of HPV according to histology					
Assay	HPV positivity	NILM	CIN1	CIN2	CIN3/ACIS	Cancer
		(n = 85)	(n = 36)	(n = 33)	(n = 139)	(n = 3)
		n (%)	n (%)	n (%)	n (%)	n (%)
HC2	Positive	27 (31.8)	20 (55.6)	30 (90.9)	132 (95.0)	3 (100.0)
Proofer	Positive	17 (20.0)	8 (22.2)	19 (57.6)	117 (84.2)	3 (100.0)
	HPV16	7 (8.2)	5 (13.9)	9 (27.3)	59 (42.4)	1 (33.3)
	HPV18	3 (3.5)	3 (8.3)	5 (15.1)	17 (12.2)	1 (33.3)
	HPV31	4 (4.7)	1 (2.8)	7 (21.2)	19 (13.4)	0 (0.0)
	HPV33	2 (2.4)	0 (0.0)	2 (6.1)	17 (12.2)	2 (67.7)
	HPV45	5 (5.9)	1 (2.8)	0 (0.0)	20 (14.4)	0 (0.0)
Anyplex	Positive	45 (52.9)	24 (66.7)	32 (97.0)	138 (99.3)	3 (100.0)
	Set A^1^	35 (41.2)	21 (58.3)	30 (90.9)	137 (98.6)	3 (100.0)
	HPV16	7 (8.2)	6 (16.7)	10 (30.3)	61 (43.9)	1 (33.3)
	HPV18	4 (54.7)	5 (13.9)	5 (15.2)	20 (14.4)	1 (33.3)
	HPV31	7 (8.2)	5 (13.9)	8 (24.2)	30 (21.6)	1 (33.3)
	HPV33	1 (1.2)	1 (2.8)	3 (9.1)	19 (13.7)	2 (67.7)
	HPV45	3 (3.5)	2 (5.6)	0 (0.0)	16 (11.5)	0 (0.0)
	HPV52	6 (7.1)	4 (11.1)	7 (21.2)	20 (14.4)	1 (33.3)
	HPV58	4 (4.7)	2 (5.6)	5 (15.2)	9 (6.5)	0 (0.0)
	other hrHPV set A^2^ pluss phrHPV set B^3^	21 (24.7)	17 (47.2)	18 (54.5)	47 (33.8)	0 (0.0)

^1^ Including HPV 16,18,31,33,35,39,45,51, 52,56,58,59,66,68

^2^ Including HPV 35, 39, 51, 56, 59, 66, 68

^3^ Including HPV 26, 53, 69, 73, 82

Abbreviations: HPV: human papillomavirus, NILM: negative for intraepithelial lesion and malignancy, CIN1: cervical intraepithelial neoplasia grade 1, CIN2: cervical intraepithelial neoplasia grade 2, CIN3: cervical intraepithelial neoplasia grade 3, ACIS: adenocarcinoma in situ, hr: high-risk, phr: possible high-risk.

Considering only the HPV tests that included genotyping (Anyplex and Proofer), there were some discrepancies in the detection of HPV16, 18, 31, 33, and 45. In women with CIN3+, four cases were negative by Anyplex for the HPV type identified by Proofer, but in three of the four cases, at least one other hrHPV type was identified by Anyplex.

The sensitivity of Anyplex set A plus set B to detect the 175 women with CIN2+ was 98.9% (95% CI 95.9–99.9); for HC2 it was 94.3% (95% CI 89.7–97.2), and for Proofer it was 79.4% (95% CI 72.7–85.2) (Table 4). All three women with invasive cancers and all with ACIS were positive for all three investigated HPV tests.

**Table 4:** Sensitivity, specificity and positive predictive value (PPV) of human papillomavirus (HPV) tests in detecting high-grade dysplasia.

HPV assays	Sensitivity (%, 95% CI)	Specificity (%, 95% CI)	PPV (%, 95% CI)
Anyplex			
CIN3+	99.3 (96.1–100)		58.3 (51.8–64.5)
CIN2+	98.9 (95.9–99.9)	43.0 (34.0–52.3)	71.5 (65.4–77.1)
Anyplex hr/phr types^1^			
CIN3+	99.3 (96.1–100)		60.5 (53.9–66.8)
CIN2+	97.7 (94.3–99.4)	48.8 (39.6–58.0)	73.4 (67.2–78.9)
Anyplex hr/phr types with medium or high viral load^2^			
CIN3+	99.3 (96.1–100)		62.4 (55.7–68.7)
CIN2+	97.7 (94.3–99.4)	54.5 (45.2–63.6)	75.7 (69.5–81.1)
Anyplex set A			
CIN3+	98.6 (95.0–99.8)		61.9 (55.3–68.3)
CIN2+	97.1 (93.5–99.1)	53.7 (44.4–62.8)	75.2 (69.1–80.7)
Anyplex set A with medium or high viral load^3^			
CIN3+	98.6 (95.0–99.8)		63.9 (57.2–70.3)
CIN2+	97.1 (93.5–99.1)	59.5 (50.2–68.3)	77.6 (71.5–83.0)
Hybrid capture 2			
CIN3+	95.1 (90.1–98.0)		63.7 (56.8–70.2)
CIN2+	94.3 (89.7–97.2)	61.2 (51.9–69.9)	77.8 (71.6–83.2)
Proofer			
CIN3+	84.5 (77.5–90.0)		73.2 (65.7–79.8)
CIN2+	79.4 (72.7–85.2)	79.3 (71.0–86.2)	84.8 (78.3–89.9)

^1^ Anyplex II HPV28 hr/phr include HPV16,18,31,33,35,39,45,51, 52,56,58,59,66,68,26,53,69,73 and 82.

^2^ Anyplex II HPV28 hr grade 2 and 3 include medium (grade 2) and high (grade 3) viral load of HPV16,18, 31,33,35,39,45,51,52,56,58,59,66,68,26,53,69,73 and 82.

^3^ Anyplex II HPV28 Set A gr 2 and 3 include medium and high viral load of HPV16,18,31,33,35,39,45,51,52, 56,58,59,66 and 68

Abbreviations: CIN2+; cervical intraepithelial neoplasia grade 2 or worse, CIN3+; cervical intraepithelial neoplasia grade 3 or worse, hr: high-risk, phr; possible high-risk

Restricting the analysis to infections with medium or high viral loads, the sensitivity of Anyplex set A plus set B to detect CIN2+ was still slightly higher than that of HC2 and significantly higher than that of Proofer, and for Anyplex set A, the sensitivity to detect CIN2+ remained high, the PPV raised slightly, and the specificity raised to the same level as HC2.

Looking at Anyplex results for HPV16 and 18 only, the PPV was high (82.5%), but only half of the CIN2 and CIN3 lesions and two of the three cancers were detected, leaving a NPV of 55.5% (Table 5).

**Table 5:** Diagnostic effect of adding high-risk human papillomavirus (HPV) types from Anyplex II HPV28 in detecting cervical dysplasia.

	HPV result according to histology					
High-risk HPV types	NILM	CIN1	CIN2	CIN3	ACIS	Cancer
	(n = 85)	(n = 36)	(n = 33)	(n = 134)	(n = 5)	(n = 3)
	n (%)	n (%)	n (%)	n (%)	n (%)	n (%)
16,18^1^						
Positive	9 (10.6)	11 (30.6)	15 (45.5)	73 (54.5)	4 (80.0)	2 (67.7)
Negative	76 (89.4)	25 (69.4)	18 (54.5)	61 (45.5)	1 (20.0)	1 (33,3)
16,18,31,33,45 ^2^						
Positive	19 (22.4)	16 (44.4)	20 (60.6)	121 (90.3)	5 (100.0)	3 (100.0)
Negative	66 (77.6)	20 (55.6)	13 (39.4)	13 (9.7)	0 (0.0)	0 (0.0)
16,18,31,33,45,52,58^3^						
Positive	26 (30.6)	17 (47.2)	25 (75.8)	129 (96.3)	5 (100.0)	3 (100.0)
Negative	59 (69.4)	19 (52.8)	8 (24.2)	5 (3.7)	0 (0.0)	0 (0.0)
16,18,31,33,35,39,45,51,52, 56,58,59 ^4^						
Positive	34 (40.0)	20 (55.6)	29 (87.9)	132 (98.5)	5 (100.0)	3 (100.0)
Negative	51 (60.0)	16 (44.4)	4 (12.1)	2 (1.5)	0 (0.0)	0 (0.0)
16,18,31,33,35,39,45,51, 52,56,58,59,68 ^5^						
Positive	35 (41.2)	20 (55.6)	29 (87.9)	132 (98.5)	5 (100.0)	3 (100.0)
Negative	50 (58.8)	16 (44.4)	4 (12.1)	2 (1.5)	0 (0.0)	0 (0.0)
16,18,31,33,35,39,45,51, 52,56,58,59,66,68 ^6^						
Positive	35 (41.2)	21 (58.3)	30 (90.9)	132 (98.5)	5 (100.0)	3 (100.0)
Negative	50 (58.8)	15 (41.7)	3 (9.1)	2 (1.5)	0 (0.0)	0 (0.0)
16,18,26,31,33,35, 39,45,51, 52,53,56,58,59,66,68, 69,73, 82^7^						
Positive	40 (47.1)	22 (61.1)	30 (90.9)	133 (99.2)	5 (100.0)	3 (100.0)
Negative	45 (52.9)	14 (38.9)	3 (9.1)	1 (0.8)	0 (0.0)	0 (0.0)

^1^ 2 hrHPV-types included in bi- and quadrivalent vaccine

^2^ 5 hrHPV-types included in Proofer

^3^ 7 hrHPV-types included in the 9-valent HPV-vaccine

^4^ 12 hrHPV-types,carcinogenic

^5^ 13 hrHPV-types included in HC2

^6^ 14 hrHPV-types present in set A in Anyplex

^7^ All 14 hrHPV-types and 5 possibly hrHPV types in Anyplex II HPV28

Abbreviations: NILM; Negative for Intraepithelial Lesion and Malignancy, CIN1; Cervical Intraepithelial Neoplasia 1, CIN2; Cervical Intraepithelial Neoplasia 2, CIN3; Cervical Intraepithelial Neoplasia 3, ACIS; Adenocarcinoma in situ

Adding more hrHPV types to the analysis lowered the PPV and raised the NPV. Analyzing Anyplex by the five hrHPV types in Proofer (HPV 16, 18, 31, 33, and 45) raised the sensitivity to detect CIN2+ to 85.1% and the NPV to 76.8%, which was comparable to values for Proofer. The same pattern was observed for CIN3+, and all cancers were detected. When we restricted the analysis to the seven hrHPV types included in the 9-valent HPV vaccine, HPV16, 18, 31, 33, 45, 52 and 58, the sensitivity of Anyplex to detect CIN2+ was 92.6% and CIN3+ 96.5%, leading to a relative sensitivity according to HC2 of 0.98 (95% CI 0.93–1.04) and 1.01 (95% CI 0.97–1.07), respectively. Specificity fell to a level comparable to HC2. Including all HPV types in Anyplex set A, increased the sensitivity of CIN2 from 75.8% to 90.9%, while sensitivity of CIN3+ had a minimal increase to 98.6%. Despite high prevalence of other hrHPV and phrHPV types in CIN2+, the majority occurred in multiple infections together with the 7 vaccine hrHPV types and contributed to little extent in the detection of CIN2+. Specificity fell as more HPV types were included in the analyses, and when including set B in addition to set A in Anyplex, specificity according to CIN2+, fell to 43.0%.

## Discussion

In our study, Anyplex, including all 28 HPV types, was a highly sensitive HPV genotyping assay, with a sensitivity of 98.9% to detect CIN2+. This is in line with recent studies that compared Anyplex and other, established genotyping tests [6, 7, 9]. When we restricted to medium and high viral loads of the hrHPV types in Anyplex set A, the specificity increased, without loss of sensitivity.

The agreement between Anyplex set A plus set B and HC2 was higher than between Anyplex set A plus set B and Proofer, which can be explained by the different HPV types included in the assays, as restricting the analysis to the hrHPV types included in Proofer led to comparable clinical test properties. The observed specificity for Anyplex set A and HC2 in our study was higher than earlier reports [15, 20]. One study of 1114 primary screening specimens evaluated the performance of Anyplex and HC2, and found an overall agreement between the two tests of 91.4% (ĸ = 0.50), where Anyplex was more sensitive in detecting HPV [8]. We found a slightly higher inter-rater agreement, which might be explained by the higher prevalence of HPV and dysplasia in our study population, with a viral load that was detectable by both tests. In addition, the guidelines for referral to colposcopy in Norway at the time of study inclusion with a delayed referral allowed for clearance of low-grade lesions, and leads to a population with a higher proportion of persistent HPV infections compared to women referred to colposcopy and biopsy after reflex HPV testing of low-grade lesions. The sensitivity of Proofer in previous studies was 70.6–78.1%, and specificity was 70.8–75.5%, which is comparable to our findings [15, 16, 20]. The relatively small variation in sensitivity and specificity observed for Proofer across different studies and study populations suggests that the design of this assay, which detects E6/E7 mRNA from the five most oncogenic HPV types, is less prone to detect transient infections and performs equally on referral populations with a somewhat divergent risk for CIN2+. However, when we restricted the analysis of Anyplex to the five hrHPV types included in Proofer (HPV16, 18, 31, 33, and 45), the differences in sensitivity and specificity between the two tests were no longer significant, indicating that the lower sensitivity and higher specificity of Proofer to detect CIN2+ is mainly due to the HPV types targeted by the assay, rather than to the type of nucleic acid detected. The agreement in the positivity rate between Proofer and Anyplex in women with CIN2+ was good for the five HPV types included in Proofer, but when restricting Anyplex to these five types, 14.9% (26/175) of CIN2+ were missed, half of which were CIN2.

Our study supports other findings that the prevalence of multiple-type HPV infections is higher in younger than older women [9]. A large study including 8182 women found that multiple-type HPV infections are more common in women with abnormal cytology [21]. We did not find a clear association between multiple-type HPV infections and dysplasia in our referral population, but this might be due to the small number of samples with normal histology. The low specificity of Anyplex set A plus set B to detect CIN2+ in our study (43.0%) would lead to a high number of unnecessary colposcopy-directed biopsies, anxiety, and examination of women. We have demonstrated that restricting the analysis to medium and high viral loads in Anyplex set A does not affect the high sensitivity of the test to detect CIN2+, but raises the specificity to the same level as that of HC2. Adding the HPV types from Anyplex set B had very little diagnostic value, as only one more case of CIN2+ (positive for HPV82) was detected. This supports that limiting a HPV detection assay to the 14 types in set A, listed in international guidelines as the minimum types to be included in a primary screening test [5], is clinically sufficient. Anyplex, including all 28 HPV types, tended to be more sensitive to detect CIN2+ and CIN3+ than HC2, but the specificity was lower. More studies in larger populations are necessary to confirm our results of an absence of benefit of Anyplex set B.

There is a potential bias regarding sensitivity, as only the tests including an internal control (Anyplex and Proofer) were re-analyzed if internal control and HPV were both negative at the same time. This was the case in only seven samples tested with Proofer, so any potentially contribution should be minimal. The sensitivity of medium and high viral loads in Anyplex set A fulfilled the criteria for HPV tests in primary screening, which stated that the clinical sensitivity of the HPV test should be not less than 90% of HC2 for ≥CIN2+ [4, 5]. Even if our comparison was performed on a referral population, the clinical sensitivity criteria defined by Meijer et al applies for women with CIN2+, and is thus relevant beyond primary screening purposes. The Anyplex II HR HPV Detection test is a newly launched test that detects the 14 hrHPV types in Anyplex set A. It is found comparable to HPV tests used in primary cervical cancer screening, like HC2 and CobasHPV Test (`Cobas 4800`), a HPV DNA test detecting the same 14 hrHPV types as Anyplex II HR HPV Detection and runs on the Cobas 4800 system (Roche Diagnostics, Meylan, France) [22–24].

The specificity of Anyplex to detect CIN2+ in our study might have been increased due to the extraction method performed on pelleted cell aliquots, which allows for the detection of HPV DNA in cell fractions, rather than free HPV viral particles in transport medium that are released during a productive transient HPV infection, as suggested by a previous study on enriched cells comparing type-specific qPCR with HC2 [25]. Pelleting cells is currently part of the manufacturer’s instructions for Anyplex. Longitudinal changes in viral loads for individual HPV types from cell-enriched samples have previously been shown to be of prognostic value for lesions that progress or regress, when viral loads were quantified per cell [26]. Even if Anyplex does not provide viral loads per cell, our results suggest a potential role of overall type-specific loads in disease severity. Further studies are needed to assess whether the semi-quantitative design of Anyplex might prove useful in determining which lesions are prone to regress or progress by individual HPV type, particularly in women with single-type HPV infections, based on changes in viral loads. However, in women with multiple-type HPV infections, the multiplex design of Anyplex might affect the amplification efficiency of every single type. In our data, Anyplex did not detect HPV45 in some women with multiple-type HPV infections that were detected by Proofer. This is in line with results from another study, in which discrepant results were encountered occasionally, mostly for HPV39 and 45 in multiple-type infections [23]. Overall type-specific results allowed the authors to conclude that Anyplex might be useful in measuring the persistence of specific HPV types. We suggest that, since Anyplex missed some HPV types in women with multiple-type infections, genotype data should be used cautiously for absolute risk stratification in women with multiple-type infections. In single-type hrHPV infections, which are most common among older women, and which are expected to be more frequently encountered in women who have received the 9-valent HPV vaccine, Anyplex may be a very useful tool in measuring the persistence of HPV infections.

When we restricted analyses to the seven hrHPV types included in the 9-valent HPV vaccine (HPV16, 18, 31, 33, 45, 52, 58), almost all CIN3+ and three of four cases of CIN2 were detected, assuming that vaccination with 9-valent vaccine will greatly reduce the burden of HPV-induced cervical disease, but emphasizing the need for a test that will differentiate between hrHPV types other than HPV16/18. Our data show that genotyping beyond type 16 and 18, including the 7-type hrHPV types 16, 18, 31, 33, 45, 52 and 58, satisfy the international criteria for sensitivity in cervical cancer screening with a relative sensitivity of Anyplex compared to HC2 for CIN2+ and CIN3+ of 0.98 and 1.01%, respectively. Our results support that extended genotyping including the 7 hrHPV-types included in the 9-valent vaccine may lead to a more precise risk-based screening and follow-up strategy of hrHPV-positive women. Anyplex is one of very few commercial tests that can genotype more than HPV16 and 18 in a simple, PCR-based, real-time assay, and can be used in measuring vaccine impact among women attending cervical cancer screening and to stratify the impact of other genotypes by risk of cervical disease in the absence of the seven most oncogenic types. HPV type replacement post-vaccination might appear. In the absence of the most frequent hrHPV types, a non-significant increase in HPV51 was seen after introducing HPV vaccination in Scotland [27, 28]. Whether this is real type replacement or the result of methodological artifacts is uncertain. Most HPV DNA genotyping tests have consensus primer PCR assays in the conserved parts of the L1 viral region, where the most prevalent HPV types, especially HPV16, might mask other HPV types present and lead to false-negative tests for these HPV types. When eliminating HPV16, the other HPV types present will not be outperformed in the PCR reactions, and thus will be unmasked. Such a diagnostic artifact was found in a study regarding HPV16 and 52 and must be considered in a post-vaccination population [29].

In 2015, HPV testing in primary screening was established in a randomized setting in four counties in Norway among women 34–69 years old, using Cobas 4800. HPV prevalence in women aged 34 years and older was 6.5%. The prevalence of CIN3+ in this group was 1.3% [30]. In a small study of 94 samples from routine screening, clinical performance of Anyplex set A was compared with that of the Cobas 4800 Test. Anyplex performed well, with almost perfect agreement (94.7%; kappa 0.89) and 100% agreement in the 20 cases of CIN2+ [31]. A larger study, including cervical specimens from almost 1300 women attending a cervical cancer screening program, enriched with 300 samples from women with abnormal cervical cytology, concluded that the analytical performance of Anyplex II HPV HR detection was non-inferior to that of HC2 and Cobas 4800 [32]. Assuming that the sensitivity of Anyplex in the study population is transferable to a screening population, and that HPV prevalence detected by Cobas 4800 is comparable to that detected by Anyplex set A, the PPV of medium and high viral load in Anyplex set A would be 20%, and NPV would be expected to reach 100%. A negative primary screening test would support safe return to screening, while a positive test would lead to substantial over-diagnosing. A triage test is therefore crucial to avoid unnecessary burdens on women and the health system. As the prevalence of hrHPV will fall due to HPV vaccination and women repeatedly screened with HPV tests, the PPV of a positive HPV test will be even further reduced. Genotyping, as well as viral loads, may prove useful as risk stratification in such low prevalence settings.

The main strength of our study is the schematic sampling, handling, and analysis of the HPV assays, performed by a few staff members at the same clinic. A single LBC specimen was used for all three HPV tests, and the two genotyping tests were performed on the same aliquot, which makes it likely that the amount of HPV was the same for all tests. Misclassification of disease was kept to a minimum, as all patients had biopsies available and histology revised.

A limitation of the study is the small number of women without CIN2+, so caution should be taken in drawing conclusions about the specificity of Anyplex in a screening population. Indeed, the PPV and NPV of a positive HPV test in our study sample will differ from observed in a screening population, as the prevalence of HPV and CIN2+ was much higher in our study. Another potential limitation is that Anyplex was performed retrospectively on DNA samples kept frozen up to 3 years at -80°C. DNA has previously been shown to be stable for up to 3 years when kept below -20°C [33], and -80°C is the temperature commonly used for long term storage of DNA in biobanks [34]. Our findings confirm the clinical sensitivity of Anyplex, as non-inferior to HC2, and thus indicate that the storage did not influence the performance of Anyplex.

## Conclusions

The hrHPV types included in Anyplex set A, similar to Anyplex II HPV HR Detection test, are sufficient to detect CIN2+ and CIN3+. When restricting Anyplex to moderate and high viral loads for the 14 hrHPV types in set A, the clinical performance was comparable to HC2 in this referral population. Restricting Anyplex to the 7 hrHPV-types 16, 18, 31, 33, 45, 52 and 58, satisfy the international criteria for the sensitivity of HPV tests in primary screening for CIN2+. Our findings indicate that extended genotyping beyond HPV16 and 18 will lead to a more precise risk stratification for CIN2+ and CIN3+ and a more precise risk based follow-up of HPV positive women. According to our results, there is no additional clinical benefit of including possible hrHPV types in diagnosing CIN2+, while a decrease in clinical specificity was observed. Anyplex, with all 28 HPV types, is not expected to add any benefit in primary cervical cancer screening, compared to the Anyplex II HR HPV Detection test or in a referral population.
